# Supplementary material for: Luteolin Alleviates Cadmium-Induced Kidney Injury by Inhibiting Oxidative DNA Damage and Repairing Autophagic Flux Blockade in Chickens
Source: Antioxidants (Basel). 2024 Apr 26;13(5):525. doi: 10.3390/antiox13050525 (PMC11117664; doi:10.3390/antiox13050525)
Supplement: Supplementary file 1 [file antioxidants-13-00525-s001.zip › antioxidants-2960925-supplementary.pdf]

antioxidants

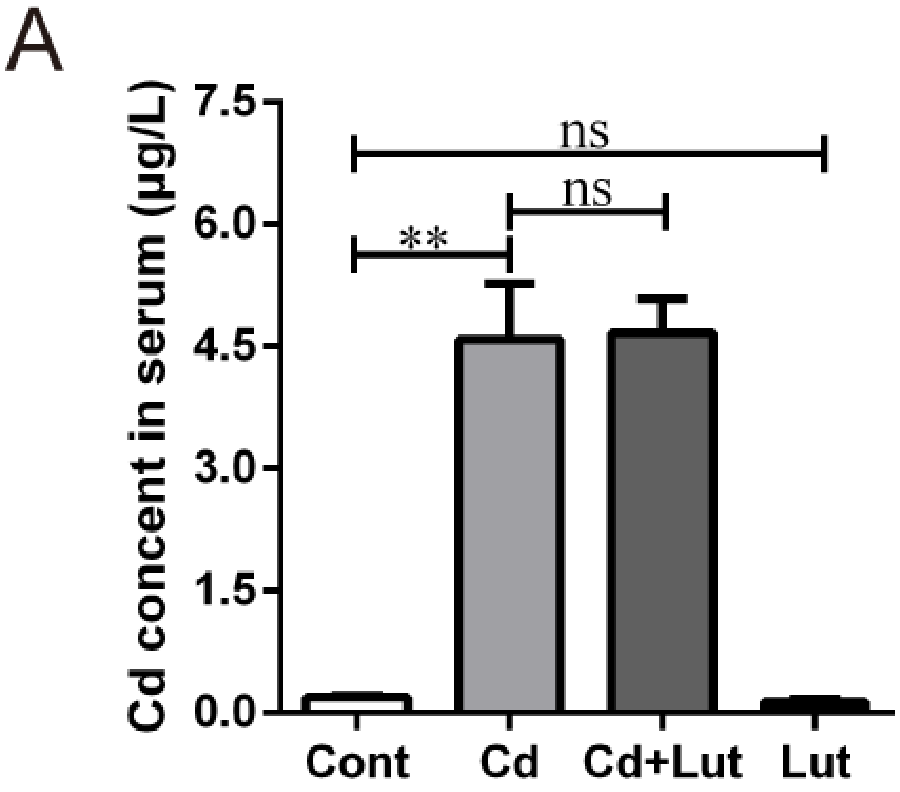

Supplementary Figure S1. Effect of Lut and/or Cd on serum Cd content. (A) Detection of serum Cd content by FAAS. Each experiment was duplicated at least three times. (ns: $P \geq 0.05$; *: $P < 0.05$, **: $p < 0.01$).
